# Modernizing Gender, Sex, and Sexual Orientation Data Through Engagement and Education

**DOI:** 10.2196/51632

**Published:** 2023-11-15

**Authors:** Lia Ginaldi, Massimo De Martinis

**Affiliations:** 1 Department of Life, Health and Environmental Sciences University of L'Aquila L'Aquila Italy

**Keywords:** data sharing, digital health systems, digital health, gender, sex, and sexual orientation, electronic health records, GSSO, health information standards, LGBT health, LGBT, policy, LGBTQIA+, gender medicine

We were pleased to read the article by Queen et al [1], which reports a sensitive, precise, and effective discussion on the need for and how to modernize the terminology of gender, sex, and sexual orientation (GSSO) in the digital health system. The authors warn us about the risks of inaccurate and potentially harmful clinical care due to the inability of most digital health systems to record, store, and manage GSSO data. Sex and gender minorities (SGMs) are emerging as relevant components of our society and are increasing numerically, but they unfortunately face health and health care inequities. Current population data remain oblivious to an accurate accounting of SGMs [2], but there has been a focus on this topic for a long time, numerous proposals have been made to overcome these shortcomings, and scientific production in this area has grown exponentially in recent years. The lack of reliable and specific data is one of the fundamental issues to focus efforts on to have reliable and valid resources to conduct studies that are specific to these population groups and help overcome health disparities. To inform treatments and policies for interventions and identify health inequities, we need larger and more diverse SGM samples that integrate multiple data sources.

Modernizing and expanding GSSO information practices may inform the development and equitable implementation of prevention and treatment guidelines for the growing lesbian, gay, bisexual, transgender, queer/questioning, and intersex (LGBTQI+) population. However, to date, a seemingly trivial issue that is fundamental and requires attention and commitment is education on inclusivity and respect for diversity. For example, numerous studies have confirmed that education on the specific health issues of LGBTQI+ populations is lacking in health curricula, and health professionals often report their shortcomings in this area. Knowledge and sensitivity to LGBTQI+ issues are fundamental in the global population and especially in each person in care pathways. If knowledge, the ability to listen and welcome, and sensitivity in data collection are lacking, any other progress in other more technical aspects is likely to be thwarted [3].

In summary, achieving equitable progress in clinical care requires addressing global disparities, which requires a multidisciplinary approach, improving the collection of health data such as GSSO information, and reducing social taboos through education [4]. Everyone, operators and users, must be given the tools to understand, be sensitive, feel involved, and participate appropriately in this process. Collaborative partnerships are the basis for responding to complex disparities in communities; positive outcomes and the codevelopment of systems and priorities can only be achieved with strong collaborative relationships. Furthermore, health equity programs, comprehensive insurance policies, gender-inclusive and gender-specific research, targeted screening, and personalized treatment programs, together with specific SGM engagement strategies [5], are crucial to eliminating health care disparities for the LGBTQI+ community.

## Abbreviations

GSSO: gender, sex, and sexual orientation
LGBTQI+: lesbian, gay, bisexual, transgender, queer/questioning, and intersex
SGM: sex and gender minority
